# Duration of viral shedding in hospitalized patients infected with pandemic H1N1

**DOI:** 10.1186/1471-2334-11-140

**Published:** 2011-05-23

**Authors:** Silvia Meschi, Marina Selleri, Eleonora Lalle, Licia Bordi, Maria B Valli, Federica Ferraro, Giuseppe Ippolito, Nicola Petrosillo, Francesco N Lauria, Maria R Capobianchi

**Affiliations:** 1 Laboratory of Virology, National Institute for Infectious Diseases 'L. Spallanzani', 292 Via Portuense, Rome, Italy; 2 Department of Epidemiology and Pre-clinical Research, National Institute for Infectious Diseases 'L. Spallanzani', 292 Via Portuense, Rome, Italy; 3 Clinical Department, National Institute for Infectious Diseases 'L. Spallanzani', 292 Via Portuense, Rome, Italy

## Abstract

**Background:**

The first influenza pandemic of the 21th century was ignited by a new strain of influenza A virus (A/H1N1pdm). Specific patient groups, including those with comorbidities, pregnant women, young children, older and immunocompromised patients, are at increased risk for serious influenza-related disease. This study was aimed at investigating the influence of clinical presentation, antiviral treatment and possible drug resistance-associated mutations, on the extent and duration of viral shedding in patients infected with A/H1N1pdm.

**Methods:**

An observational study was performed, based on retrospective review of clinical and laboratory records of patients who were hospitalized for A/H1N1pdm infection at the National Institute for Infectious Diseases "L. Spallanzani", Rome, Italy, between April 24 and December 31, 2009. Among 119 hospitalized patients, 39 were selected for a post hoc analysis, based on the availability of serial nasopharyngeal swabs samples and related information.

**Results:**

Eleven out of the 39 study patients (28.2%) presented with pneumonia; 29 (74.4%) received antiviral treatment. Patients with pneumonia were significantly older than patients without pneumonia. The mean values of viral RNA concentration were not significantly increased in patients with pneumonia, but a significant increase in the duration of viral shedding was observed as compared to patients without pneumonia. In patients receiving antivirals, the viral RNA concentration was significantly reduced in comparison to untreated patients at days 4-5 after symptom onset, while the overall duration of viral shedding was only marginally affected. A significant correlation between duration of viral shedding and time elapsed between symptom onset and therapy start was observed, with a significant reduction of days of viral shedding when therapy was initiated within 2 days of symptoms appearance. No known drug resistance mutations were detected in patients with prolonged viral shedding.

**Conclusions:**

Our results show that severe respiratory illness is associated with delayed virus clearance in patients with A/H1N1pdm infection. Antivirals caused an early reduction of viral load, but only marginally affected the overall duration of shedding. Prolonged shedding was not associated with the emergence of strains carrying known drug-resistance mutations.

## Background

In March/April 2009, a new pandemic influenza A virus (A/H1N1pdm) emerged in Mexico and spread rapidly via human-to-human transmission, originating the first pandemic of the 21th century [1].

A recent overview of the clinical aspects of A/H1N1pdm infection has been published by WHO [2]. Specific patient groups, including those with comorbidities, pregnant women, young children, older patients and individuals with compromised immunity, are at higher risk than the general population for serious influenza-related disease and hospitalization [3].

Prolonged course of illness and severe complications seem to be associated with a delayed presentation for care, higher initial viral loads in upper respiratory samples [4] and emergence of antiviral-resistance [5].

Previous studies have shown that in uncomplicated illness, A/H1N1pdm titers in nasopharyngeal swabs (NPS) peak on the day of symptom onset and gradually decline thereafter [6], while in severely ill patients higher viral load and slower decline of viral shedding are observed [2]. In addition, a more protracted viral replication has been described in adults hospitalized with seasonal influenza as compared to outpatients with uncomplicated illness [7].

Oseltamivir, one of the neuraminidase inhibitors (NAI), has been the drug of choice for treatment of A/H1N1pdm infection; in addition, 2 other drugs were available: zanamavir, which is a US Food and Drug Administration-approved inhaled NAI [8], and peramivir, which is an intravenous NAI released under an emergency use authorization during the pandemic [9]. In case of an uncomplicated illness, the early use of oseltamivir is usually associated with prompt clearance of infectious influenza A/H1N1pdm virus from the upper respiratory tract [10]. The rapid curtailment of active viral replication in the respiratory tract, determined by antiviral therapy, may reduce the duration of hospitalization [11] and the risk of progression to severe disease [12,13]. Moreover, the prophylactic administration of oseltamivir to subjects exposed to influenza A/H1N1pdm has been reported to reduce the rate of symptomatic infection, although it does not always prevent the infection [14]. In patients treated with oseltamivir, younger age (< 13 years) has been associated with prolonged viral shedding, while clinical severity has been associated with a higher viral load in the upper respiratory tract [15].

The aim of present study was to establish the influence of clinical presentation and of antiviral treatment, as well as of possible drug resistance-associated mutations, on the extent and duration of viral shedding in patients infected with influenza A/H1N1pdm. To this aim we performed an observational study, by retrospectively reviewing the clinical and laboratory records of patients infected with influenza A/H1N1pdm who were hospitalized at the "L. Spallanzani" National Institute for Infectious Diseases in Rome, Italy, between April 24 and December 31, 2009.

## Methods

### Patients

During the study period (April 24 - December 31, 2009), 119 patients were hospitalized at the "L. Spallanzani" National Institute for Infectious Diseases, of whom 54 presented with pneumonia. Hospitalization was not a general hallmark of clinical severity, as the clinical management of patients changed during the study period, in compliance with the modifications in the National policy of pandemic influenza management. In particular, during the pandemic containment response, patients with laboratory confirmed infection were hospitalized not only on the basis of clinical severity, but also to isolate them from the general population and limit the spread of a new and potentially serious infectious disease. From August 2009 onwards, patients were hospitalized if they developed potentially serious medical conditions or if the exacerbation of their underlying chronic illnesses or severe symptoms were considered to be unmanageable at home [16].

For the hospitalized patients, the local hospital policy for pandemic influenza management included the assessment of viral shedding at subsequent time points during the hospitalization period, in order to tailor the individual isolation measures, and to monitor the efficacy of treatment in patients receiving antivirals.

Clinical and laboratory records of patients presenting at the hospital for A/H1N1pdm infection, for whom serial nasopharyngeal swab samples had been collected during hospitalization, were reviewed.

This study was exempt from ethical review, and no approval for use of data was necessary, since it was based on a retrospective chart review and analyses were performed on an anonymized database. To this respect, the local policy was complying with the international policy recently reviewed [17], and with the current Italian legislation.

### Specimen collection and laboratory investigation

During the study period, the Virology Laboratory of "L. Spallanzani" provided diagnostic service for patients referred by the local admission department and by other regional hospitals. Nasopharyngeal swabs, placed in viral transport medium, were sent to the laboratory within 12 hours of collection. Upon arrival in the laboratory, the samples were divided in two aliquots and stored frozen at -80°C if not processed immediately.

From all samples, nucleic acids were purified with the QIAamp Virus BioRobot MDx kit (QIAGEN, Valencia, CA, USA) on an MDx BioRobot platform. A/H1N1pdm diagnosis was based on the positivity to the real-time reverse trascriptase-polymerase chain reaction (RT-PCR) established by the Centers for Diseases Control and Prevention (CDC), specific for detection and characterization of A/H1N1pdm virus [18]. Both pan-influenza A (targeting M gene), swine influenza A-specific (targeting NP gene) and A/H1N1pdm-specific (targeting H gene) primer/probe sets were used; in parallel, the constitutive gene RNAseP was amplified, as positive control of extraction and amplification.

For quantitative evaluation, a reference standard curve was prepared by using a reference influenza A/H1N1pdm virus preparation supplied by the European Network for Diagnostics of Imported Viral Diseases (ENIVD), containing a known genome-equivalent concentration (3.55 × 10^6 ^genomes/ml). For each sample, the M gene cycle threshold value was plotted against the calibration curve and the values expressed as copies/mL of the starting material. The detection limit of this assay is 80 copies/mL, as established by Probit analysis in our laboratory.

The presence of drug resistance mutations was established by sequencing the neuraminidase (NA) gene. Specifically, nucleic acids were amplified by in house methods using One-Step qRT-PCR system (Invitrogen, Carlsbad CA, USA) to yield partial (forward: 5'-gacaacagtataagaatcggttc-3' position 307-329 nt; reverse: 5'-acccacggtcgattcgagcc-3', position 892-911) or full-length sequences (forward: 5'-atgaatccaaaccaaaagataataacc-3', position 1-27; reverse: 5'-gtcaatggtaaatggcaactcagc-3', position 1380-1403) of the NA gene. Sequencing was performed on an automated ABI Prism 3130 instrument (Applied Biosystems, Foster City CA, USA) by using the Big Dye3.1 cycle sequencing kits provided by the same manufacturer. All the sequences have been deposited in GenBank with the following accession numbers [GenBank: CY052089; CY052076; CY052087; CY055373; CY055390; CY052092; from CY064903 to CY064921; CY064937 and CY064938].

### Statistical evaluation

Influenza viral RNA concentration (expressed in copies/mL) were Log10 transformed for statistical analysis. For most variables, descriptive statistics, such as mean ± standard deviation (SD), median with interquartile range (IQR), and proportion (%), were calculated. An arbitrary value of 1.9 Log10 copies/ml was assigned to PCR-negative samples when comparing the mean values from the various groups of patients. The student's t, Mann-Whitney U, and χ^2 ^(or Fisher's exact test when applicable) tests were used for univariate analysis when appropriate. A two-tailed *p*-value < 0.05 was considered significant. Statistical analyses were performed using the SPSS software, version 16.0 (SPSS).

## Results

### Viral load according to the time lapse between symptom onset and presentation for diagnosis

**Figure 1:**
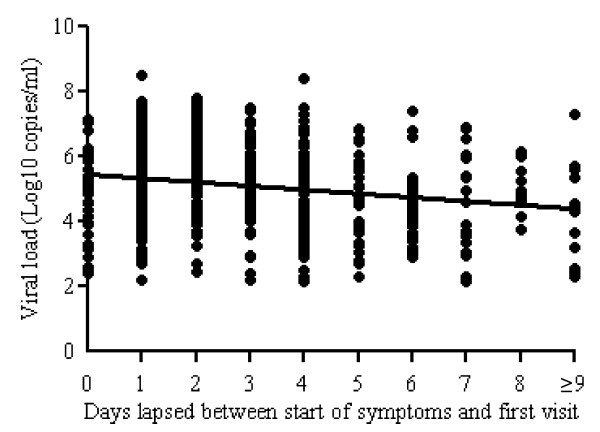
**Correlation of initial viral load with the duration after symptom onset in 533 patients**. The analysis was performed on 533 NPS collected during the first visit and sent to the Virology Laboratory of the National Institute for Infectious Diseases "L. Spallanzani", Rome, Italy for laboratory diagnosis (r = -0.203, p < 0.0001).

During the whole observation period, nasopharyngeal swab samples from 2,609 patients were analysed by real-time RT-PCR, to establish an A/H1N1pdm diagnosis; among them, 870 samples (33.3%) were A/H1N1pdm-positive. Among 533 patients (20.4%) for whom the time of symptom onset was available, the mean viral load in the diagnostic sample was 5.2 ± 1.3 Log10 copies/ml. As shown in Figure 1, viral load at first presentation correlated negatively with time since symptom onset (r = -0.203, p < 0.0001); at day 2 and ≥9, the mean viral load values were highest (5.7 ± 1.2, p < 0.001) and lowest (4.2 ± 1.5, p = 0.020), respectively.

Before August 2009 all patients with influenza like illness, mostly with mild symptoms, were referred for laboratory diagnosis, while, from August onwards, a diagnosis was only required for severely ill patients; we may therefore assume that the samples collected in the first period largely represented mild patients, while those collected later represented more severe cases. When comparing the mean viral load values observed in the diagnostic samples collected before or after August 2009, no significant differences were observed (5.37 ± 1.20 vs. 5.23 ± 1.26, respectively, p = 0.220), suggesting that the severity of clinical presentation did not affect viral load values at first clinical observation. Subsequent studies were performed on better characterized patients to further investigate this point.

### Effect of clinical severity and antiviral treatment on the extent and duration of viral shedding in hospitalized patients

Among 119 patients hospitalized during the observation period at the "L. Spallanzani" National Institute for Infectious Diseases, serial respiratory samples were available for 39 patients, of whom 11 (28.2%) had pneumonia. The demographic and clinical characteristics of these patients are shown in Table 1, where patients are grouped according to the presence or absence of pneumonia. The vast majority (9/11) of patients with pneumonia already showed this complication at presentation. Two patients developed pneumonia after hospitalization; they were coinfected with *H. influenzae *and *M. tuberculosis*, respectively, and both received early antiviral treatment.

**Table 1:** Demographic and clinical characteristics of 39 patients hospitalized at INMI

		With pneumonia	Without pneumonia	p value^a^
	**Patients: n. (%)**	11 (28.2)	28 (71.8)	

	Age in years: median (IQR)	51 (41-63.5)	26 (18.5-35.5)	< 0.001
**Demographics**	Age ≥65: n. (%)	2 (18.2)	1 (3.6)	
	Sex: M/F	4/7	19/9	

	Asthma	2 (18.2)	3 (10.7)	
	Immunosuppression^b^	3(27.3)	3 (10.7)	
	Diabetes	3 (27.3)	4 (14.3)	
**Underlying conditions: n. (%)**	Obesity	3 (27.3)	1 (3.6)	
	Chronic respiratory failure	1 (9.1)	3 (10.7)	
	Pregnancy	0	1 (3.6)	
	Cardiopathy or hypertension	3 (27.3)	3 (10.7)	
	Others	2 (18.2)	0	

	Headache	2 (18.2)	14(50.0)	
	Asthenia or Malaise	4 (36.4)	24 (85.7)	0.009
	Arthromyalgia	5 (45.5)	15 (53.6)	
**Presenting symptoms: n. (%)**	Chills	2 (18.2)	4 (14.3)	
	Conjunctivitis	0	4 (14.3)	
	Cough	10 (90.9)	21 (75.0)	
	Sore throat	5 (45.5)	16 (57.1)	
	Coryza	1 (9.1)	14 (50.0)	0.046
	Dyspnea	7 (63.6)	6 (21.4)	0.035
	Dehydration	1 (9.1)	6 (21.4)	
	Gastrointestinal symptoms	4 (36.4)	8 (28.6)	

**Day of illness at presentation: median (IQR)**		2 (1-3)	2.5 (1-4)	

**Respiratory virus coinfections: n. (%)**		0	3^c ^(10.7)	

	**Antiviral**	10 (90.9)	19 (67.9)	
	Days of therapy: median (IQR)	7 (7-16.5)	6 (5-8)	0.011
**Therapy: n. (%)^d^**	Patient starting therapy within 48 h	5 (45.5)	11 (39.3)	
	**Antibiotic/antimycotic**	10 (90.9)	11 (39.3)	0.004
	**Steroid**	2 (18.2)	2 (7.1)	

^a ^p values ≥0.05 have been omitted.

^b ^HIV, n = 5; hairy cells leukaemia, n = 1.

^c ^Human respiratory syncytial virus type A, n = 2; human rhinovirus, n = 1.

^d ^Unless otherwise specified.

Patients with pneumonia were significantly older than those without pneumonia (median 51 vs. 26 years, p < 0.001) and more likely to present with dyspnoea (63.6% vs. 21.4%, p = 0.035) but less likely to have asthenia or malaise (36.4% vs. 85.7%, p = 0.009) and coryza (9.1% vs. 50%, p = 0.046). No significant gender difference was observed among the two groups (Table 1).

In 11 patients with pneumonia, no additional complications were observed, while, among patients without pneumonia, 6 of 28 (21.4%) presented one of the following complications: exacerbation of chronic obstructive pulmonary disease (COPD) or of asthma (n = 4, 14.3%); petechial rush (n = 1, 3.6%); elevated ALT (n = 1, 3.6%).

Twenty-nine out of 39 patients received antiviral treatment with oral oseltamivir, 25 (86.2%) at standard dosage (i.e. 75 mg twice/day) [19] while 4 (13.8%) at increased dosage (i.e. 150 mg twice/day), according to the medical judgment of the treating physician; in the late phases of hospitalization, intravenous zanamivir replaced oral oseltamivir in 2 patients with pneumonia, initially treated with standard (n = 1) or increased dosage (n = 1) of oseltamivir.

Oseltamivir treatment was initiated on the same day of symptom onset (day 0) for 1 (3.5%), at day 1 for 8 (27.6%) and at day 2 for 7 (24.1%) patients; for the 13 remaining patients (44.8%) therapy start was in a range of 3-13 days from symptom onset. Median therapy duration was 7 days (IQR, 7-16.5 days) and 6 days (IQR, 5-8 days) in patients with and without pneumonia, respectively (p = 0.011). Ten out of 11 patients with pneumonia (90.9%) received antiviral treatment.

Mean viral load values at presentation were not significantly different in patients grouped according to clinical severity, i.e. presence or absence of pneumonia (5.01 ± 1.66 vs. 5.49 ± 1.35, p = 0.360).

The time course of viral load in these patients was further analysed with respect to time from symptom onset, according to presence of pneumonia and administration of antivirals. Three or more respiratory specimens for each patient were analysed (164 available results from a total of 175 collected NPS), sampled over a median time lapse of 7 days (IQR, 4-14 days) and 5 days (IQR, 3-7 days) since symptom onset for patients with and without pneumonia, respectively (p = 0.003).

**Figure 2:**
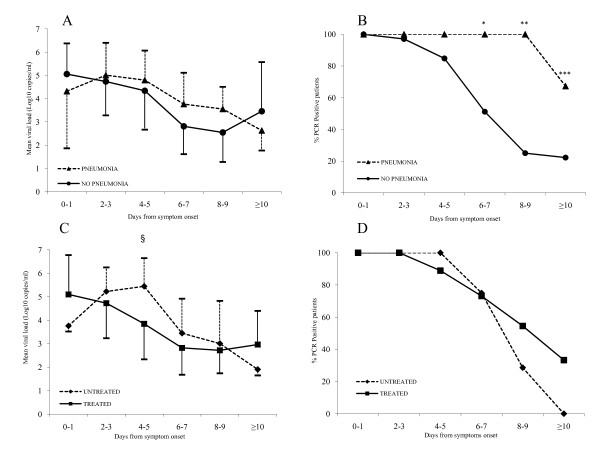
**Time course of viral load and duration of viral shedding according to presence of pneumonia and antiviral treatment**. Panels A and C: mean values and standard deviation (represented by bars) of influenza A/H1N1pdm in NPS at various time points after symptom onset; panels B and D: proportion of samples positive to influenza A/H1N1pdm PCR according to time from symptom onset. Panels A and B: patients with pneumonia vs. without pneumonia; Panels C and D: treated vs. untreated patients. In panel A the number of samples collected at days 0-1, 2-3, 4-5, 6-7, 8-9 and ≥10 were (for patients with pneumonia) 3, 8, 8, 9, 5, and 21 and (for patients without pneumonia) 8, 21, 30, 7, 14 and 10. In panel C the number of samples collected at days 0-1, 2-3, 4-5, 6-7, 8-9 and ≥10 were (for treated patients) 9, 24, 24, 23, 13 and 29, and (for untreated patients) 2, 5, 14, 13, 6 and 2. * p ≤ 0.010, ** p ≤ 0.010, *** p ≤ 0.010; §: p = 0.002.

As shown in Figure 2A, the time course of viral load in NPS from patients with and without pneumonia was similar. Consistently, the overall mean viral load values were not significantly different in the 2 groups (3.7 ± 1.5 vs. 3.8 ± 1.7 Log10 copies/mL, respectively). However, when considering the duration of viral shedding, virus RNA was detectable for longer time in the NPS from patients with pneumonia as compared to patients without pneumonia (mean number of days with PCR-positive NPS since the symptom onset: 15.4 ± 9.0 vs. 7.5 ± 3.4, p = 0.002; mean number of days with PCR-positive NPS since the first visit:12.1 ± 7.9 vs. 4.8 ± 3.8 days, p = 0.003). Consistently, from day 6 onward, the proportion of patients with PCR-positive NPS was significantly higher in patients with pneumonia as compared to those without pneumonia. In fact, as shown in Figure 2B, at days 6-7, 8-9 and ≥10 the proportion of PCR-positive patients was 100%, 100% and 67.3% for patients with pneumonia, and 51.2%, 25% and 22.2% for patients without pneumonia, p < 0.001. On the whole, 100% of patients with pneumonia and 66.7% of patients without pneumonia had PCR-positive samples after day 6 from start of symptoms, approaching statistical significance (p = 0.063). To be noted, one patient with pneumonia, despite oseltamivir and zanamivir treatment, had PCR-positive NPS until day 34 post symptom onset.

Analysis of viral load and duration of shedding was performed also according to antiviral therapy. When considering the overall mean values of viral load, these were significantly lower in treated compared with untreated patients (3.6 ± 1.6 vs. 4.2 ± 1.7 Log10 copies/mL, p = 0.038). In Figure 2C the time course of viral loads in patients grouped in treated vs. untreated is shown. In untreated patients, the viral load steadily increased from baseline up to days 4-5 after symptom onset, then declined. At days 4-5 the mean viral load value in untreated patients was significantly higher than that of treated patients (5.4 ± 1.2 vs. 3.9 ± 1.5 Log10 copies/mL, respectively; p = 0.002). When considering the length of viral shedding in untreated vs. treated patients, there was a tendency to shorter duration in the treated group, although the difference was not statistically significant (7.7 ± 1.8 vs. 10.2 ± 7.2 days, p = 0.378, Figure 2D).

**Figure 3:**
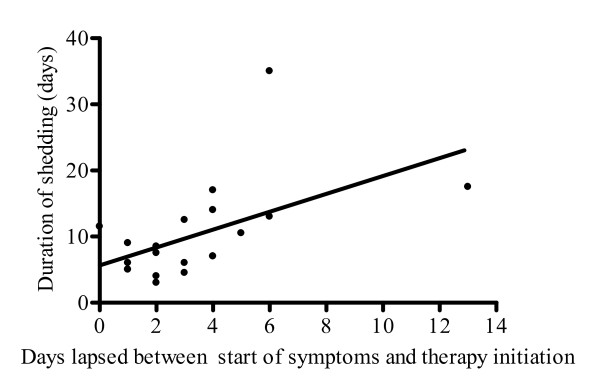
**Correlation of duration of viral shedding with interval between symptom onset and therapy start**. The analysis was performed on data from 20 patients treated with inhibitors of NA, who reached undetectable PCR during the observation period (r = 0.531, p = 0.016).

Among treated patients, a positive correlation was observed between the overall duration of viral shedding and the days elapsed between symptom onset and start of antiviral therapy (Figure 3, r = 0.531, p = 0.016). Consistently, the mean duration of shedding in patients who started treatment within 2 days of therapy initiation was significantly shorter than in those who started therapy at later times (6.6 ± 2.7 vs. 13 ± 8.5, p = 0.042, respectively).

Table 2 reports the analysis of the association of a number of factors with the prolonged shedding (positivity of NPS samples to influenza PCR for > 6 days) of A/H1N1pdm. As can be seen, among the considered factors, presence of any complication (pneumonia, exacerbation of COPD or asthma, petechial rash, elevated ALT), pneumonia and duration of antiviral administration, but not administration per se, were significantly associated with prolonged viral shedding. These data support that the deciding factor for prolonged shedding was, in fact, the presence of pneumonia; however, due to the small number of cases, it was not possible to apply a multivariate analysis to specifically address this point.

**Table 2:** Positivity of nasopharyngeal swabs for influenza virus RNA 6 days after symptom onset according to patient characteristics

Variable	N. of patients positive for viral RNA 6 days after symptom onset/total (%)	p value^a^
**Sex**		
**Male**	15/21 (71.4)	
**Female**	13/16 (81.3)	

**Immunosuppression^b^**		
**Present**	6/6 (100)	
**Absent**	22/31 (71)	

**Underlying lung disease^c^**		
**Present**	7/8 (87.5)	
**Absent**	21/29 (72.4)	

**Any complication^d^**		0.005
**Present**	17/17 (100)	
**Absent**	11/22 (50)	

**Any complication excluding pneumonia**		
**Present**	6/6 (100)	
**Absent**	11/20 (55)	

**Pneumonia**		0.02
**Present**	11/11 (100)	
**Absent**	17/26 (65.4)	

**Antiviral treatment**		
**Yes**	21/28 (75)	
**No**	7/9 (77.8)	

**Time from symptom onset to initiation of treatment**		
**≤ 2 days**	10/15 (66.7)	
**> 2 days**	11/13 (84.6)	

**Antiviral treatment regimen^e^**		
**standard**	4/5 (80)	
**intensified**	17/23 (73.9)	

**Antiviral treatment duration**		0.02
**≤ 5 days**	3/8 (37.5)	
**> 5 days**	18/20 (90)	

**Antibiotic/antimycotic treatment**		
**yes**	18/20 (90)	
**no**	10/17 (58.8)	

**Steroid treatment**		
**yes**	4/4 (100)	
**no**	24/33 (72.7)	

^a ^by chi-square test; p values ≥0.05 have been omitted.

^b ^HIV or hairy cells leukaemia.

^c ^COPD or Asthma.

^d ^Pneumonia, exacerbation of COPD or asthma, petechial rash, elevated ALT.

^e ^Standard treatment: oseltamivir 75 mg twice/day; intensified treatment: increased dosage of oseltamivir or oseltamivir+zanamivir.

### Analysis of antiviral resistance

Since the emergence of resistance-associated mutations is among the factors associated with prolonged viral shedding, particularly in patients treated with antivirals [20], we decided to establish the mutational pattern for those patients for whom sufficient sample volume was available to perform the sequence analysis. In particular, NA sequencing analysis of pandemic influenza virus was achieved for 13 treated patients (8/13 with shedding duration > 6 days, of whom 3 were immunocompromised, 2 with HIV and 1 with leukaemia; 12 with oseltamivir alone, 1 sequentially treated with oseltamivir and zanamivir). The total number of analysed NPS from 13 treated patients was 16, with a median sampling time of 2.88 (range 0-15) days from therapy onset. For comparison, 5 untreated patients (4 with shedding duration > 6 days) were also analysed. No mutations known to be associated with NAI resistance were detected.

## Discussion

In this study, the concentration of influenza A/H1N1pdm RNA in NPS was evaluated in relation to the time from start of symptoms, considering all patients with confirmed infection whose samples had been sent for diagnosis to the Virology Laboratory of "L. Spallanzani" Institute.

In addition, the influence of clinical severity and of antiviral treatment on the extent and duration of viral shedding was evaluated in a group of hospitalized patients, for whom sequential NPS samples had been analysed. The clinical findings of these patients were not uniformly severe, since, at least at the beginning of pandemics, most patients with confirmed infection were hospitalized.

Concerning the first point, the data shown in Figure 1, representing a cross-sectional evaluation of the viral load of patients at first presentation, indicate that the highest initial values of H1N1/pdm RNA concentration were observed in patients presenting for influenza diagnosis on day 2 from start of symptoms. A significant trend to decrease according to the distance between the symptom onset and first sampling was observed, in line with the reports from other groups [6,21].

Concerning the second point, in patients serially sampled during their hospitalization period, a progressive decline of viral load was observed in those who received antivirals, while in untreated patients an increase of viral load was observed during the first 5 days, followed by a decrease (Figure 2C). This result is in apparent contrast with the trend to a progressive decline of viral load at first presentation shown in Figure 1. However, the data from this figure represent a cross-sectional analysis of first viral loads detected in patients at diagnosis, and therefore are not directly comparable to those from Figure 2, where the time course in patients serially sampled is reported.

In apparent contrast with data from other authors [15], in the present study mean viral load values at presentation were not significantly different in patients grouped according to clinical severity, i.e. presence or absence of pneumonia. However, and more importantly, patients with pneumonia showed a delayed viral clearance. In fact, about 85% of patients without pneumonia had influenza-negative samples at days 8-9 after symptom onset (Figure 2B), and 78% after this time point, while a significantly lower proportion of patients with pneumonia were negative at the same time points. It is not possible from our results to determine whether the viral genomes detected in the upper respiratory tract of patients after the initial stages of the infection correspond to actually infectious virions. Lower positive rates for virus culture, compared with RT-PCR assay are commonly observed [4], and other studies have reported that infectious virus may be detected after the resolution of fever and sometimes after the completion of therapy [22].

Although there is the possibility of selection bias, due to the fact that patients with more severe clinical course**s **received protracted care, and were sampled for longer periods compared to those with milder symptoms, these findings are in agreement with a number of previous reports. For instance, To et al. [21], have shown a slower decline in viral shedding in patients with severe conditions as compared to the mild disease groups, and, recently, Li et al. [15], have shown similar findings considering treated patients only.

All the factors significantly associated with prolonged viral shedding, i.e. presence of any complication, pneumonia and duration of antiviral administration (Table 2), are strictly interconnected, suggesting that the deciding factor for prolonged shedding is, in fact, the presence of pneumonia. However, because of limited number of cases, it was not possible to apply multivariate analysis to identify the effect of one of the variables (pneumonia and early antiviral treatment) adjusting for the other.

It is reasonable to assume that the delayed clearance observed in severe cases could be secondary to a worse control of viral replication, due to a less effective innate and adaptive immune response, as recently suggested by our group [23].

Another possible reason for the prolonged virus replication in the upper respiratory tract may be the emergence of viral strains with reduced susceptibility to NAI. In fact, resistant strains have been observed after prolonged administration of antivirals. These resistant strains typically contain a single H275Y substitution in the viral NA gene, and are mostly detected in specimens obtained from patients with a severely compromised immune system and from patients who received oseltamivir, but still had persistent viral replication [5,20]. The emergence of drug-resistant pandemic influenza strains is not a major public health concern so far, as the majority of the influenza A H1N1/pdm viruses are oseltamivir-susceptible, while oseltamivir-resistant strains remain infrequent and are still sensitive to zanamivir [24]. However, resistant strains have been also isolated in untreated patients, including known or suspected cases of person to person transmission [25]. In our study, the emergence of resistant strains as a possible factor underlying the prolonged viral shedding may be considered negligible, as no mutations known to be associated to either oseltamivir or zanamivir resistance were detected in either treated or untreated patients. To this respect, our findings are in line with recent observations by other authors. For instance Fleury et al. reported prolonged influenza A/H1N1pdm shedding not associated with the emergence of resistance mutation in the viral NA gene in 2 severely ill patients [26].

The absence of a link between the emergence of resistant strains due to therapy administration and the prolonged viral shedding from the upper respiratory tract of hospitalized patients is further supported by the absence of statistically significant differences in the persistence of PCR positivity in treated vs. untreated patients (Figure 2D), despite a significant reduction of viral load observed at days 4-5 from symptom onset in treated patients (Figure 2C). The lack of significant differences in the persistence of PCR positivity in treated vs. untreated patients is in agreement with data from other studies carried out on seasonal influenza strains [27], and in apparent contrast with other studies, showing a significant shortening of the duration of viral shedding in A/H1N1pdm -patients exposed to NAI [28,10]. In another study conducted only in treated patients, more prolonged viral shedding was observed in subjects < 13 years of age than in older patients [15]. In our study we did not evaluate the association between age and delayed virus clearance, since the age of our patients was rather homogeneous, and our case series did not include young patients (Table 2), because our Institute is not a pediatric referral hospital.

Our data indicate a positive correlation between the overall duration of viral shedding and days elapsed between symptom onset and start of antiviral therapy (Figure 3). These results are in agreement with the widely held concept that early initiation of antiviral therapy is important in order to obtain an effective control of the viral replication, and in turn, to shorten the duration of symptoms [10].

Our study has some limitations: first, the number of patients is rather small, and the lack of significance of some differences may be due to insufficient size of the compared groups; due to the limited number of patients, it was not possible to apply multivariate analysis to the factors associated with prolonged shedding; furthermore, the collection of samples was not prospectively planned, so the sampling was not conducted at fixed time points. Several studies have indicated that viral shedding in the upper respiratory tract is shorter and not always representative of lower airway shedding [2,26]. In the cases under study, serial bronchoalveolar lavage (BAL) samples were analysed only for the patient with leukaemia, who showed severe pneumonia and received enhanced antiviral treatment. In this patient the BAL returned negative earlier than NPS (day 21 vs. day 34), but no conclusion can be drawn from this anecdotal observation.

## Conclusions

In conclusion, our data indicate that in patients with influenza A/H1N1pdm infection, severe clinical presentations such as pneumonia are significantly associated with a prolonged virus shedding. In addition, the administration of antivirals significantly reduced viral load at early times from the start of treatment, but only had a marginal effect on the duration of viral shedding. The positive correlation between the overall duration of viral shedding and days elapsed between symptom onset and start of antiviral therapy underscores the necessity of a timely initiation of antiviral therapy to obtain an effective control of viral spread.

Further investigation is mandatory to better understand the significance of prolonged shedding that seems to occur in patients with severe presentation. No link between the emergence of resistant strains due to therapy administration and prolonged viral shedding from the upper respiratory tract of hospitalized patients was observed in this study. The delayed virus clearance observed in severe patients is consistent with impaired innate immunity, as was observed in a recent study by our group [23]. This may be relevant for the duration of contagiousness, and may have clinical/pathogenetic significance.

## Financial Competing interests

The authors declare that they have no competing interests.

## Authors' contributions

SM, MS carried out the laboratory investigations, participated to collection of clinical records and to the statistical evaluation of the results and drafted the manuscript. EL, LB contributed to the laboratory investigations. MBV carried out the sequencing work and contributed to the manuscript writing. FF participated to the collection of clinical records and to the statistical analysis. GI supervised the study design. NP was responsible for the clinical management of most of the hospitalized patients included in the study. FNL supervised the clinical management of the patients, participated to the study design and to the manuscript preparation. MRC supervised the laboratory work, participated to the study design and to the manuscript preparation. All authors read and approved the final manuscript.

## Pre-publication history

The pre-publication history for this paper can be accessed here:

http://www.biomedcentral.com/1471-2334/11/140/prepub
